# Complement Has Brains—Do Intracellular Complement and Immunometabolism Cooperate in Tissue Homeostasis and Behavior?

**DOI:** 10.3389/fimmu.2021.629986

**Published:** 2021-02-25

**Authors:** Natalia Kunz, Claudia Kemper

**Affiliations:** ^1^ Complement and Inflammation Research Section (CIRS), National Heart, Lung and Blood Institute, Bethesda, MD, United States; ^2^ Institute for Systemic Inflammation Research, University of Lübeck, Lübeck, Germany

**Keywords:** complement/complosome, metabolism, T cells, IFN-γ, IL-10, brain, behavior, evolution

## Abstract

The classical liver-derived and serum-effective complement system is well appreciated as a key mediator of host protection *via* instruction of innate and adaptive immunity. However, recent studies have discovered an intracellularly active complement system, the complosome, which has emerged as a central regulator of the core metabolic pathways fueling human immune cell activity. Induction of expression of components of the complosome, particularly complement component C3, during transmigration from the circulation into peripheral tissues is a defining characteristic of monocytes and T cells in tissues. Intracellular complement activity is required to induce metabolic reprogramming of immune cells, including increased glycolytic flux and OXPHOS, which drive the production of the pro-inflammatory cytokine IFN-γ. Consequently, reduced complosome activity translates into defects in normal monocyte activation, faulty Th1 and cytotoxic T lymphocyte responses and loss of protective tissue immunity. Intriguingly, neurological research has identified an unexpected connection between the physiological presence of innate and adaptive immune cells and certain cytokines, including IFN-γ, in and around the brain and normal brain function. In this opinion piece, we will first review the current state of research regarding complement driven metabolic reprogramming in the context of immune cell tissue entry and residency. We will then discuss how published work on the role of IFN-γ and T cells in the brain support a hypothesis that an evolutionarily conserved cooperation between the complosome, cell metabolism and IFN-γ regulates organismal behavior, as well as immunity.

## Introduction

**Figure 1:**
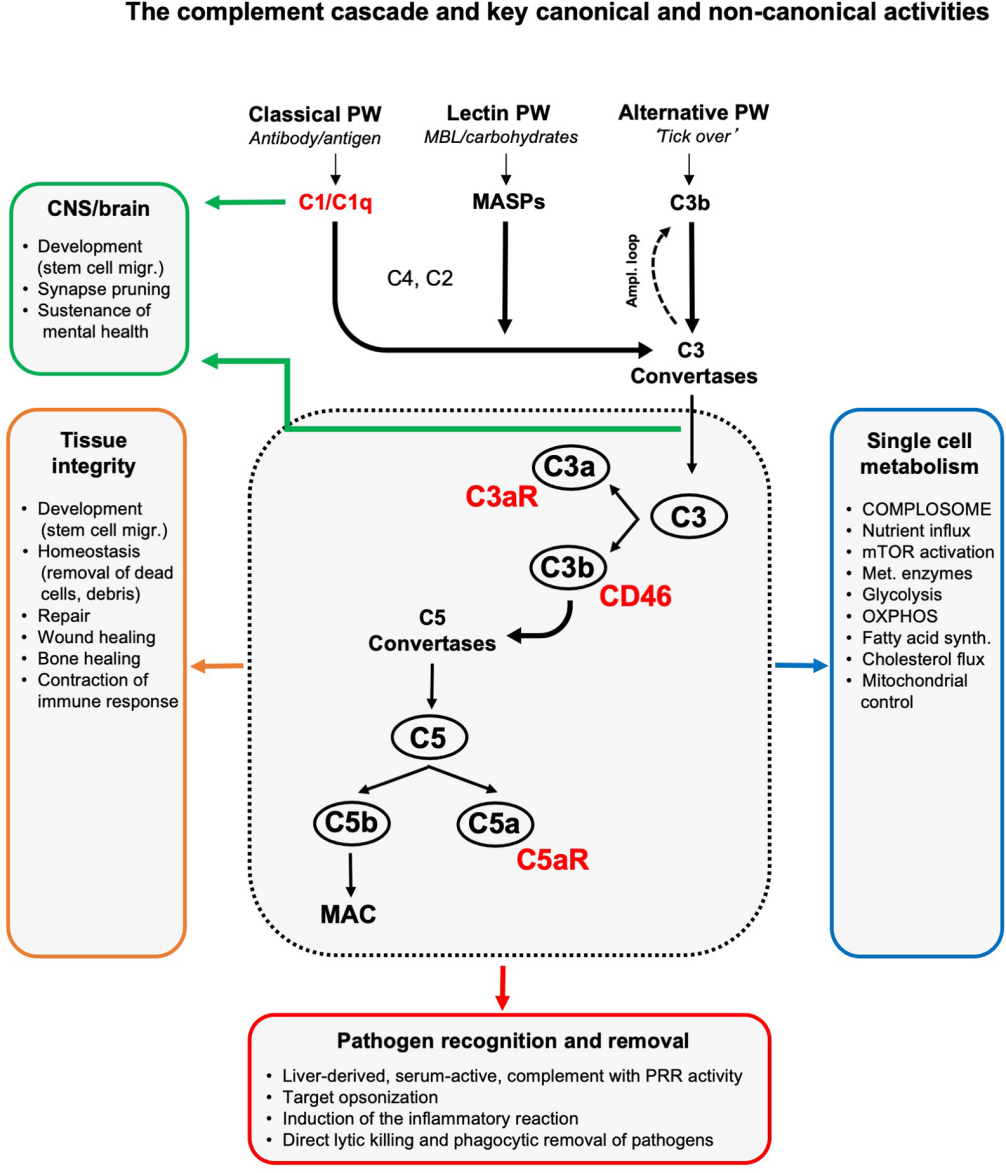
The complement cascade and key canonical and non-canonical activities. Liver-derived complement circulating in blood and the lymph can be activated through three pathways (of which two have PPR activity): the classical, lectin and alternative pathway. The initial deposition of C3b or C3b(H_2_O), generated by the so-called ‘tick over’ reaction, onto an activating target also initiates a positive feedback amplification loop. The formation of C3 convertases triggered by serum complement pathogen recognition then induces cleavage activation of C3 into C3a and C3b. Subsequent C5 convertase formation activates C5 into C5a and C5b, with surface-bound C5b triggering the formation and insertion of the lytic MAC into pathogen or target membranes. C3b opsonizes and tags targets for removal by scavenger cells. Receptors for complement activation fragments, such as the anaphylatoxin receptors C3a receptor (C3aR) and C5aR1/2, and the C3b receptor CD46 mediate the induction of the general inflammatory reaction (vasodilation, neutrophil, mast cell and macrophage influx and activation, etc.) which together then lead to pathogen removal as key canonical complement function. (Mostly) local complement activities are major contributors to generative and re-generative tissue processes as they mediate normal tissue development, homeostasis and repair upon insult. This non-canonical role for complement emerged as a major driver of normal central nervous system (CNS) and brain development and physiology (see text for details). The unexpected presence of a cell-intrinsic and intracellularly activated and functioning complement system, the complosome, connects the complement system now also tightly with the control of single cell metabolism (see text for details). To keep the schematic concise and accessible, we focused on the key complement components and functions discussed in this review and have, thus, omitted many other complement factors (receptors, regulators, etc.) and activities as well as the proteases that can activate C3 and C5 in a C3/C5 convertase-independent fashion (5). Ampl. loop, amplification loop; MAC, membrane attack complex; MASPs, mannose binding lectin-associated serine proteases; migr., migration; mTOR, mammalian target of rapamycin; OXPHOS, oxidative phosphorylation; PRR, pattern-recognition receptor; PW, pathway; synth., synthesis.

The complement system is broadly acknowledged as a pillar of innate immunity. Complement belongs to the evolutionarily old group of pattern-recognition receptors (PRRs) that also include toll-like receptors (TLRs), inflammasomes, and RIG-I like receptors (RLRs). Complement is commonly recognized as a liver-derived and serum-effective system, composed of over 50 fluid phase or cell-bound proteins that together form the first line of defense in the detection and removal of invading pathogens. In the absence of infection, serum complement is mostly comprised of inactive pro-enzymes. These become rapidly activated in a cascade-like fashion when any of the three complement activation pathways – the classical, lectin, or alternative pathway – are triggered by the presence of pathogens. All three activation pathways culminate in cleavage and activation of the core complement components C3 and C5 into C3a and C3b, and C5a and C5b, respectively (1–3). Immunoglobulins and C3b are the host’s major opsonins. Deposition of C3b onto pathogen surfaces triggers removal of C3b-ospinized microbes by C3b receptor-expressing scavenger cells. Deposition of C5b onto pathogens initiates assembly of the terminal pathway, generation of the pore-forming membrane attack complex (MAC) and ultimately pathogen lysis. C3a and C5a generated during C3 and C5 cleavage are anaphylatoxins. The engagement of their respective G protein-coupled signaling receptors (GPCRs), the C3a receptor (C3aR), C5aR1 or C5aR2, on innate and adaptive immune cells induces immune cell migration, induction of cell-specific effector functions and the general inflammatory reaction (4) (**Figure 1**). The immunological importance of circulating complement is demonstrated by the fact that deficiencies in key complement components cause recurrent infections [e.g (6, 7), reviewed in depth in (8, 9)]. Importantly, complement is not only vigilant in the recognition of pathogen-associated patterns (PAMPs) and protection against infections: similar to the other PRR systems (10–14), complement senses danger-associated patterns (DAMPs) displayed on stressed or apoptotic cells and injured or infected tissues and initiates the safe removal of such noxious entities. In consequence, perturbations in complement’s DAMP-sensing activities underly a range of autoimmune diseases [e.g. (15), reviewed in depth in (16–18)]. Moreover, complement also plays an active role in tissue development and homeostasis as its local activity is also a requirement for coordinated cell migration during tissue development (19), tissue repair and regeneration upon injury (including lens repair and bone healing) (20, 21). An unexpected observation, that complement produced in the central nervous system (22, 23) is required for normal brain development by controlling synaptic pruning (24), has recently initiated an entirely new and ongoing research area, namely the physiological and pathophysiological roles of complement in neurology.

Several additional recent discoveries further support the growing notion that complement occupies a more prominent position in normal cellular physiology and homeostasis than previously thought. Firstly, complement activation and function is not confined to the extracellular space, but occurs intracellularly in immune cells (2). Secondly, intracellular complement [which we have coined the complosome to set it apart from the liver-derived complement system (25)] is an unexpected central player in the regulation of single-cell nutrient flux and metabolic pathways in T and other immune cells (1, 26). Thirdly, transcriptional induction of complosome components has emerged as a key characteristic of immune cells occupying tissues (27).

Classically, the presence of cytokine-producing immune cells in one of the most specialized tissues, the brain, was considered a ‘warning sign’ for ongoing brain inflammation (28). However, recent advances suggest a clear beneficial role for presence of innate and adaptive immune cells in and around the brain for its normal development and function (29). Particularly, the metabolic state and interferon-producing capacity of ‘brain-residing’ immune cells impact heavily on brain activities determining behavior (30–32). These unexpected observations suggest an evolutionarily conserved, non-immunological, role for cytokines and immune cells in the central nervous system (CNS). In this overview article, we will first concisely summarize what is known about the complosome with regard to single cell metabolic reprogramming required for generation of the prototypical pro- and anti-inflammatory cytokines IFN-γ and IL-10 by T cells – with an eye on immune cell tissue entry and residency during infections. We will then review the newest insights into the non-canonical roles of IFN-γ, IL-10 and T cells in the brain. Finally, we propose ways in which the complosome-metabolism-cytokine axis may play a role beyond immunity but impart unexpected CNS signals that control brain homeostasis and host behavior.

## The Complosome-Metabolism-IFN-γ Axis

Despite liver-derived complement in serum being firmly established as sentinel and protector against pathogens that successfully breach host protective barriers (33, 34), functionally important extrahepatic sources of complement have long been observed (35). Immune cells, in particular, can generate complement components in an intrinsic manner to support their functional activities. For example, antigen presenting cells (APCs) and T cells can secrete C3 and C5, which are then activated extracellularly. The stimulation of complement receptors on APCs and T cells by such locally produced complement activation fragments strongly support APC antigen presentation and T cell activation during cognate APC-T cell interactions (36–38). These elegant studies already indicate a close functional connection between complement and IFN-γ, as mice lacking C3aR or C5aR1 on T cells and/or APCs display a predominant defect in T helper (Th) type 1 immunity. The recent identification of the complosome as a key driver of the metabolic pathways controlling lymphocyte survival and Th1 effector activity provide the first clues that the complement-IFN-γ relationship has metabolic roots.

Cellular metabolic pathways are fundamentally intertwined with immune cell development, survival, homeostasis and specialized functions. In principle, stored energy and molecular building blocks derived from catabolic reactions sustain housekeeping functions in most resting immune cells. Activated and proliferating cells, on the other hand, predominantly engage anabolic pathways to build biomass (nucleotides, proteins, and lipids). Work on monocytes, macrophages and T cells over the past decade have highlighted the mechanisms by which immune cell activities are enabled and controlled by specific cell-intrinsic adaptation of the metabolic machinery (39). For example, non-activated T cells in circulation or in tissues rely mostly on energy generated from oxidative phosphorylation (OXPHOS) (40, 41). T cell receptor activation and co-stimulation, triggered by infections, prompts rapid upregulation of nutrient transporters allowing influx of amino acids (AAs), fatty acids, and glucose (42–44). AA influx is sensed by the central metabolic checkpoint kinase mammalian target of rapamycin (mTOR), which induces increased glycolysis, OXPHOS and lipid synthesis to support proliferation and differentiation into effector cells (45–47). Simultaneous (micro)environmental signals, such as from cytokines and other growth factors, fine-tune TCR-triggered metabolic pathways to direct differentiation into different CD4^+^ T helper subsets (Th1, Th2, Th9, Th17, etc.), regulatory T (Treg) cells, and cytotoxic CD8^+^ T cells (CTLs) – which are all characterized by discrete metabolic profiles (40, 48). Importantly, key metabolic pathways are not simple providers of energy in the form of adenosine triphosphate (ATP), nicotinamide adenine dinucleotide (NAD^+^), etc. but serve additional, ‘non-canonical’, functions. For example, the glycolytic enzyme pyruvate kinase M2 (PKM2) interacts with the transcription factor (TF) signal transducer and activator of transcription (STAT)-3 in the nucleus to drive Th17 responses (49). Certain fatty acids can directly bind to and activate the TFs peroxisome proliferator-activated receptors (PPARs), which are members of the nuclear hormone receptors family, and are key orchestrators of T helper lineage differentiation (50). Clearance of infection removes the TCR stimulus, reducing metabolic activity and returning cells to a quiescent state. Previously activated or memory T cells show an altered epigenetic landscape, with a more poised or open chromatin state, compared to naïve cells, at a number of genetic loci encoding metabolic proteins. These epigenetic imprints allow rapid energy delivery for cellular recall responses in the case of pathogen re-encounter (51–53).

**Figure 2:**
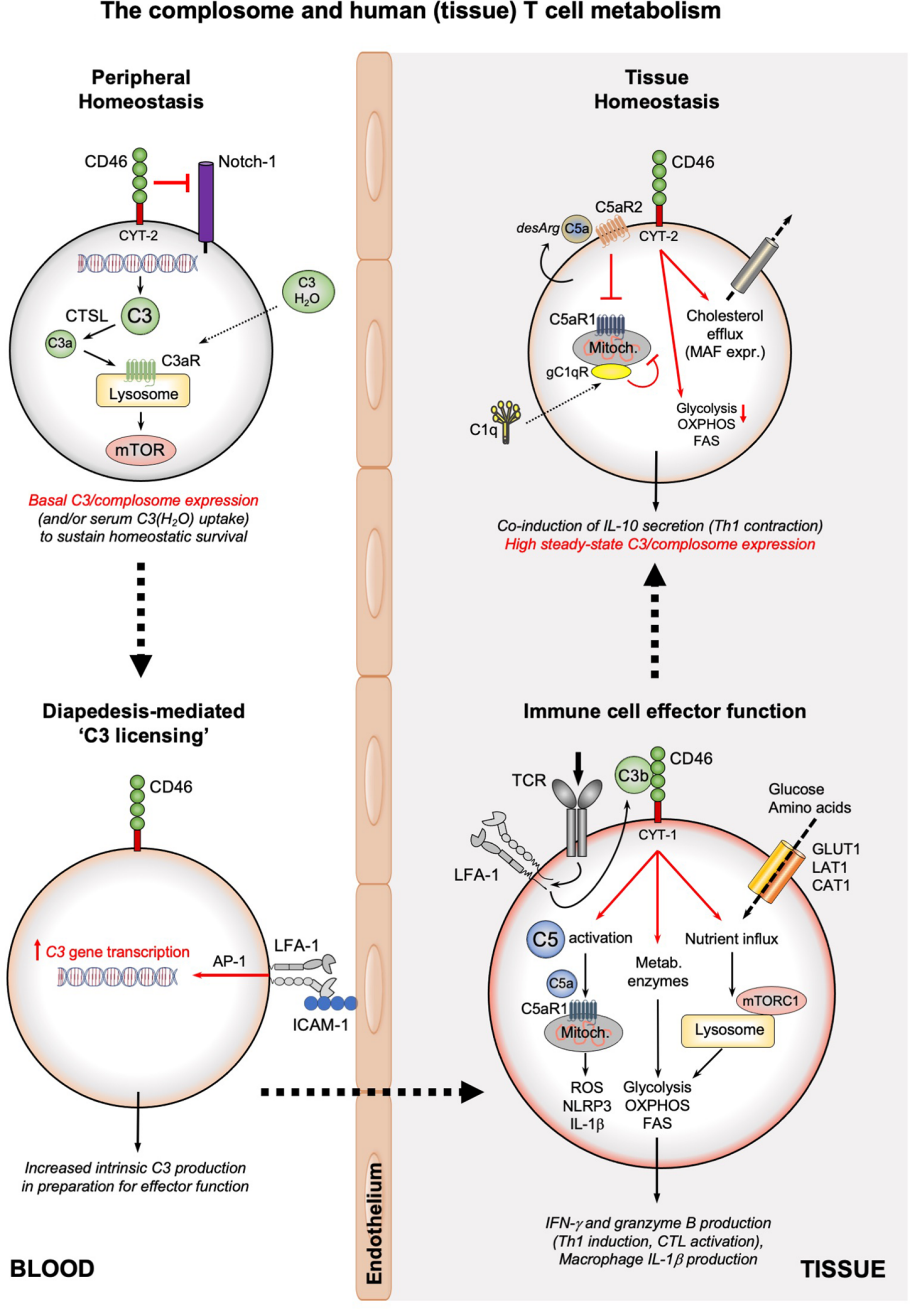
The complosome and human (tissue) T cell metabolism. The survival of circulating, non-activated CD4^+^ and CD8^+^ T cells is maintained by low-level expression of C3 (or uptake of C3(H_2_O)) that is continuously cleaved by CTSL into intracellular C3a which supports tonic mTOR activation through the lysosomal C3aR. In addition, CD46 surface expression prevents activating Notch-1 stimulation. Diapedesis of T cells (or interaction with APCs presenting cognate antigen, not shown) into tissue involves engagement of LFA-1 on T cells by ICAM-1 on endothelial cells and induces high C3 gene expression in an AP-1-depenent fashion. Timely incoming TCR signals induce rapid translocation of intracellular C3b to the cell surface, where it engages CD46. CD46 signaling triggers three key metabolic events: the γ-secretase-processed intracellular CYT-1 domain of CD46 translocates to the nucleus (not shown) where it induces expression of nutrient transporters (GLUT1, LAT1, and CAT1) as well as LAMTOR5-driven mTORC1 assembly at the lysosomes; CD46 activation induces increased expression of metabolic enzymes, including fatty acid synthase, GAPDH, etc.; CD46 also strongly augments activation of intracellular C5 pools with the intracellularly generated C5a stimulating mitochondrial C5aR1 that drives ROS production and NLRP3 inflammasome activation in CD4^+^ T cells. Together, these events underly the high levels of glycolysis, OXPHOS and ROS production needed specifically for the induction of IFN-γ production and granzyme B expression and hence protective Th1 and CTL effector responses in tissues. Of note, macrophages also rely on the LFA-1-mediated process for ‘C3 licensing’ to produce normal amounts of IL-1β upon TLR stimulation (not shown). The complosome also contributes to the safe metabolic ‘shut-down’ of human T cell immunity and prevention of tissue pathology as the CD46 intracellular domain CYT-2 reduces glycolysis and OXPHOS while supporting cholesterol efflux and MAF expression, all required for immune-suppressive IL-10 co-induction and demarcating the Th1 contraction phase. This contraction program is further supported by autocrine engagement of the repressive C5aR2 on the T cell surface (*via* intrinsic C5a-desArg), which reduces C5aR1 activity. C1q, taken up by the activated T cell can reduce mitochondrial activity (in CD8^+^ T cells) *via* a C1qR-dependent unknown mechanism. A defining feature of T cells (and macrophages, not shown) in tissues is their high steady-state expression of the complosome. CAT1, cationic amino acid transporter; CTSL, cathepsin L; FAS, fatty acid synthase/synthesis; GLUT1; glucose transporter 1; ICAM-1, intercellular adhesion molecule 1; LFA-1, lymphocyte function-associated antigen 1; LAT1, large neutral amino acid transporter 1; MAF, cMaf musculoaponeurotic fibrosarcoma oncogene homolog; mTOR, mechanistic target of rapamycin; mTORC1, mechanistic target of rapamycin complex 1; NLRP3, NLR family pyrin domain containing 3; OXPHOS, oxidative phosphorylation; ROS, reactive oxidation species; TCR, T cell receptor.

The complosome emerges as important conductor of metabolic events particularly underlying Th1 and CTL responses (1, 26, 27, 54). Specifically, human CD4^+^ and CD8^+^ T cells in circulation have stores of C3 (mostly found in the endoplasmatic reticulum (ER) and lysosomes) which are continuously cleaved by the protease cathepsin L (CTSL) into C3a and C3b. In CD4^+^ T cells, such intracellularly generated C3a engages the lysosomal C3aR and induces tonic mTOR activation that sustains T cell survival in the periphery (**Figure 2**). Upon TCR activation, intracellularly generated C3a and C3b fragments shuttle rapidly to the cell surface where they engage surface-expressed C3aR and the C3b receptor CD46. CD46 (membrane cofactor protein, MCP) is a ubiquitously expressed and multifunctional transmembrane protein (55). CD46 prevents unwanted complement deposition on host cells as a cofactor for the proteolytic inactivation of C3b and C4b by the complement protease Factor I (56). CD46 is also an entry receptor for a range of pathogenic viruses and bacteria (57), mediates sperm-egg fusion (58), and is an important co-stimulator during T cell activation (59). While CD46 is expressed on all nucleated human cells, mice and rats lack CD46 on somatic cells and an exact functional, complement-derived, homolog to human CD46 has so far not been identified in rodents (60). Therefore, the signaling events and functions controlled by CD46 are human-specific. CD46 is expressed by most cells as four distinct isoforms that arise *via* differential splicing of a single gene. The isoforms differ in the level of *O*-glycosylation of the extracellular CD46 protein portion, and bear either one of two distinct intracellular domains, termed CYT-1 and CYT-2 (giving rise to CD46^CYT-1^ and CD46^CYT-2^ isoforms) (55, 61). Both tails can trigger specific signaling events in a broad range of cell types (62). Resting T cells generally express predominantly the CD46^CYT-2^ isoform. TCR-driven autocrine engagement of CD46 by T cell-derived C3b sets several events into motion: it causes the rapid upregulation of the CD46^CYT-1^ isoform, subsequent metalloproteinase (MMP)-mediated processing and release of the extracellular CD46 portion (63), and processing of the intracellular tails of CD46 by the γ-secretase complex (61). These tails translocate to the nucleus and function as transcriptional regulators, inducing a number of genes, of which many are nutrient transporters, metabolic sensors/regulators and enzymes. For example, CD46^CYT-1^ is critically required for increased transcription of *SLC2A1*, which encodes the glucose transporter 1 (GLUT-1), *SLC7A5*, which encodes the large neutral amino acid transporter 1 (LAT-1), and *LAMTOR5* (LAMTOR5 is a scaffolding protein that supports mTOR complex 1 (mTORC1) assembly at the lysosomes) (26) (**Figure 2**). Cumulatively, these events induce the very high levels of nutrient influx, glycolysis and mTORC1 activation that are needed for metabolically demanding IFN-γ and Th1 responses (64). CD46^CYT-1^ signaling further enhances a Th1 phenotype by increasing expression of IL-2 receptor α-chain (CD25), resulting in assembly of the high affinity IL-2 receptor, necessary for optimal Th1 responses (65). CD46 engagement during T cell activation also mobilizes intracellular stores of complement C5 by inducing cleavage into C5a and C5b. Intracellular C5a generated in human CD4^+^ T cells binds C5aR1 on mitochondria and augments production of reactive oxygen species (ROS). ROS trigger assembly of the NLR family pyrin domain containing 3 protein (NLRP3) inflammasome, which catalyzes processing and secretion of mature IL-1β. IL-1β controls the duration of Th1 responses in an autocrine/paracrine fashion by maintaining secretion of IFN-γ (1).

Human CD8^+^ T cells also harbor a complosome and TCR-triggered autocrine CD46 engagement drives nutrient influx, IFN-γ production, and cytotoxic activity in these cells (54). Interestingly, in CTLs, aside from strong OXPHOS induction, CD46 is also a potent inducer of fatty acid synthesis. Although CD8^+^ T cells express NLRP3, the inflammasome is not required for normal IFN-γ secretion or cytotoxicity in CTLs (54), indicating important but distinct contributions of complosome-inflammasome crosstalk in CD4^+^ and CD8^+^ T cells.

The central role of CD46^CYT-1^ in the successful induction of IFN-γ production by human T cells is underpinned by the fact that patients deficient in CD46 or its ligand (C3/C3b) have impaired Th1 responses (at least early in life) and suffer from recurrent infections (65). Of note, CD46 and C3-deficient patients have T cells that proliferate normally and intact Th2 immunity, further supporting the notion that the complosome is particularly key to Th1 biology (65, 66).

Complement is by no means a simple supporter of inflammation but a key orchestrator of immunity, especially T cell homeostasis. For example, CD46 on resting and circulating T cells prevents T cells activation and maintains the quiescent state by binding and sequestering the Notch-1 ligand, Jagged-1. This prevents a productive Notch-1-Jagged-1 interaction that would normally trigger Th1 differentiation. Conversely, when T cells are activated by TCR triggering, MMPs are induced and mediate shedding of CD46, which releases the “brake” on Notch-1 that subsequently supports Th1 differentiation (65, 67).

CD46 is also key to the timely resolution of successful Th1 responses. This is a critical phase of immune responses and central to the host’s health because it limits tissue pathologies accompanying uncontrolled and/or prolonged T cell activation (68). A key mediator in the restraint of Th1 responses is the anti-inflammatory and immuno-suppressive cytokine, IL-10. The central role for IL-10 in Th1 control is demonstrated by the finding that *Il10* ^–/–^ mice clear some infections more rapidly than wild type animals due to augmented Th1 immunity, but then succumb to uncontrolled tissue inflammation (69, 70). CD46, together with signals from the IL-2 receptor, induces Th1 contraction by triggering co-expression of IL-10 in expanded human Th1 cells (71). The exact signals downstream of the IL-2R or CD46 that drive IL-10 production are currently not fully understood. They do, however, involve the reversion of CD46 isoforms back to a predominant CD46^CYT-2^ form, leading to downregulation of GLUT-1, LAT-1 and LAMTOR5, limitation of nutrient influx, reduced mTORC1 activity, and the general return of the T cell to a metabolically OXPHOS-dependent and quiescent state (26, 72, 73). In addition, CD46 simultaneously induces the cholesterol biosynthesis pathway and cholesterol flux, both of which are required for c-MAF-driven IL-10 expression in contracting Th1 cells (74). Not surprisingly, perturbations in CD46 signals are connected with several autoimmune states characterized by Th1 hyperactivity, such as rheumatoid arthritis (RA), systemic lupus erythematosus (SLE), multiple sclerosis (MS), and scleroderma (71, 75–77).

Similar to CD46, T cell-intrinsic C5 also contributes to negative control of Th1 cells. C5a is processed by carboxypeptidase M to C5a-desArg, the des-Arginized’ form of C5a. C5a-desArg engages C5aR2, the inhibitory C5a receptor, in an autocrine/paracrine fashion. C5aR2-mediated signals then reduce ROS production, NLRP3 inflammasome activation, and IL-1β secretion (1) (**Figure 2**).

Thus, the complosome and specifically CD46 are integral components of metabolic signatures that denote Th1 homeostasis, Th1 effector function and Th1 contraction. Further, the complosome operates in close collaboration with other intracellular danger sensing systems, such as the inflammasomes. Finally, since CD46 is only present in humans, there are clear divergent roles for complosome components between mice and men in Th1 biology.

## The Complosome as an Emerging Key Characteristic of Immune Cells in Tissues

Our current knowledge about the functions of the complosome are mostly been derived from T lymphocytes. It is clear, however, that the complosome is present in a broad range of immune and non-immune cells (25, 78). For example, human monocytes lacking intrinsic *C3* gene expression fail to develop into normally functioning dendritic cells (79) and C3-deficient macrophages produce significantly less pro-inflammatory cytokines (27). Further, human lung epithelial cells utilize storages of C3 to protect themselves from stress-induced cell death (80). Understanding how the complosome is regulated will potentially enable therapeutic modulation of this system. Our search for upstream regulators of *C3* and *C5* gene transcription has led recently to the unexpected observation that cell-intrinsic transcription of complement genes is one of the most significantly biological pathway distinguishing immune cell populations in healthy human lung tissue compared to the same cells in circulation (27). Furthermore, the induction of *C3* gene expression in immune cells (including CD4^+^ and CD8^+^ T cells and macrophages) is dependent on signals delivered by the integrin leukocyte adhesion factor 1 (LFA-1) (**Figure 2**). LFA-1 is an integrin composed of the two distinct integrin protein chains αLβ2 or CD11a/CD18 (genes *ITGAL* and *ITGB2)* and plays major roles in immune cell migration and activation (81). LFA-1 expressed on immune cells is engaged by intercellular adhesion molecule 1 (ICAM-1) expressed on endothelial cells during diapedesis or cognate antigen presenting cell (APC)-T cells during priming. LFA-1-driven *C3* gene transcription is, at least in part, dependent on the TF activation protein 1 (AP-1) (27, 82). This ‘C3 licensing’ (which remains stable for about 12 h) increases intracellular stores of C3 in anticipation of antigen encounter in tissues. Timely danger sensing transducing TCR or TLR signals then induce the increased intracellular processing of C3 into C3a and C3b which enable nutrient flux, metabolic reprogramming, and ultimately cell-specific effector function (see above). This scenario elegantly allows maintenance of survival-sustaining C3 levels in naive (and central memory) T cells upon (re)transmigration through high endothelial venules (2) and gives them time to scan APCs in lymph nodes for cognate antigen. Similarly, circulating effector memory T cells are ‘pre-loaded’ with C3 protein while surveying non-inflamed peripheral tissue and can respond rapidly when re-encountering pathogens (83). The requirement of LFA-1 as a major inducer of immune cell *C3* gene expression is underpinned by the finding that patients with mutations in CD18, who suffer from leukocyte adhesion deficiency type 1 (LAD-1), have defective Th1 and CTL responses, as well as a reduction in monocyte IL-1β secretion and are severely immune-compromised. Importantly, LAD-1 patients have low level *C3* gene expression and C3a generation, which sustain homeostatic survival, but fail to generate the augmented C3 needed for induction of productive cellular effector functions (27). The compromised Th1 phenotype of LAD-1 patients was originally thought to be caused by a combination of suboptimal TCR signaling (84–87), reduced formation of the peripheral supramolecular activation cluster (pSMAC) (88), and/or muted Notch-1 activity in CD4^+^ T cells (89), as these events require upstream LFA-1 stimulation. Our own work suggests that failure of autocrine CD46 engagement *via* increased intrinsic C3 expression is an additional major defect in LAD-1 cells. In fact, a dysfunctional LFA-1 – C3 – CD46 axis may be the common perturbation upstream of the functional changes previously observed in LAD-1 T cells: CD46 signals *per se* mimic key functions attributed to LFA-1 during T cell activation, including synergy with TCR signals, modulation of Notch-1 activity and induction of glycolysis and OXPHOS (26, 27, 81, 90). Moreover, CD46-deficient and LAD-1 patients both generate functional Th2 and Th17 responses, suggesting a functional interdependence of these molecules specifically for Th1 induction. However, this productive cooperation can also go wrong and contribute to hyper-inflammation. We found that T cells that transmigrated into the inflamed synovium in an LFA-1-dependent fashion from patients with RA display increased *C3* and *IFNG* gene expression when compared to their circulating blood cells. Moreover, T cell *C3* mRNA expression distinguished inflamed RA from uninflamed RA and performed better as a biomarker of disease severity compared to *IFNG* expression (27).

The complosome is clearly an integral part of the inflammatory functions of Th1 cells, CTLs, and macrophages. There is accumulating evidence that intracellular complement may also partake in T cell memory formation and/or maintenance of immune cell tissue residency (**Figure 2**). For example, LFA-1-mediated signals underlie normal central T cell memory formation (91, 92) and sustain retention of tissue-resident memory T cells after infections are cleared (93, 94). These observations, together with the unexpected finding that tissue-occupying immune cells in the healthy lung express a virtually complete intracellular complement system, supports the notion of a novel role for the complosome in tissue immune cell biology. Tissue-resident T cells are characterized by a specific and distinct metabolic program that is largely dictated by environmental cues provided by the (specialized) tissue (95). Intriguingly, glycolysis and OXPHOS are differently impacted by LFA-1 and CD46-mediated signals in naive and memory T cells. This suggests that the LFA-1 – C3 axis has the capacity to implement the discrete metabolic programs underlying diverse T cell subpopulations, including tissue-resident T cells (96).

Importantly, the ‘tissue complosome’, which allows tissue-resident immune cells to remain vigilant and able to respond rapidly to stimulation (**Figure 2**), can clearly be further engaged during (re)infections: by analyzing bulk RNA-seq data from lung tissues of patients with SARS-CoV2 infection and uninfected controls we have found that five of the 36 (14%) enriched pathways induced by SARS-CoV2 were annotated as complement pathways. Among the cells responding most strongly to the virus with augmentation of complosome related genes were airway epithelial cells and macrophages, followed by T cells (97). A specific role for the complosome in sustaining protective tissue immunity at barrier surfaces, such as evident for the lung, is consistent with the acknowledged role of complement as a pathogen sentinel. Moreover, the function(s) of the complosome in tissue biology may, again, extend beyond mediation of immune responses to pathogens. For example, complosome-inflammasome crosstalk in human CD4^+^ T cells restrains Th17 responses in the gut during inflammation and reduces tissue injury (1).

Overall, recent studies point toward a central role for the complosome in immune cell tissue activity and/or residency; however, as intriguing as this idea is, it currently remains to be formally explored.

## A Role for the Complement–Metabolism–Cytokine Axis in Normal Brain Physiology?

The brain, as one of the most complex and distinctive tissues/organs, has always held a particularly strong fascination for humans, and indication for interest in neuroscience dates back to the 5^th^ century BC (98). Neurological research in the last decade unexpectedly identified local complement production as a key ‘ingredient’ of a normally developed and functioning CNS. Similarly, the ‘non-canonical’ activities of certain cytokines, among those IFN-γ and IL-10, as well as the physiological presence of innate and adaptive immune cells in and around the brain, have emerged as central players to normal brain function. In the following, we will initially review what is currently known about the roles of complement, IFNs and IL-10, and of T cells in the healthy CNS. We will finalize this article with a hypothetical model suggesting how these separate effector systems may have engaged in functional co-operation during evolution for the benefit of physiological brain activity and host behavior.

### Complement and the Brain

The brain regulates fundamental physiological functions ranging from heart rate and blood pressure to complex higher order cognitive functions, such as thought and behavior. The brain is thus a central node for maintenance of body homeostasis and function. Exciting work over the recent past have identified the complement system as an unexpected, but key, system integral to normal brain functioning. There are numerous excellent and in-depth reviews available on this subject [for example (22, 99–101)] and we will therefore summarize here only the key findings.

Local complement production (mostly by astrocytes and glial cells) in the brain has been observed for many years and has almost exclusively been viewed as inflammatory, destructive and detrimental to brain function (102). This view was challenged by early studies employing classic animal systems, including zebrafish, *Xenopus* and rodents, which hinted at a particularly close relationship between complement and the neuronal system. Today, we know that complement is physiologically produced in the brain (22, 24) and that it is required for normal brain development and activity (103). The majority of core complement components (for example, C3, C4, and C5), as well as complement receptors (anaphylatoxin receptors, C1q receptors, etc…), are expressed at particularly high levels in the neuronal crest during development in model species, where they support migration of neuronal crest cells and the foundation for a normal CNS (104). Perturbations in local complement activity causes aberrant embryonic neuronal tube formation with different degrees of severity (105). Among the most impactful recent observations surrounding the new roles for complement in the CNS was the finding that complement plays a role here not only prenatally but also postnatally. Specifically, C1q sustains neuronal survival in mice *via* control of apoptosis during neural stress (106), and is required for synaptic pruning. Synaptic pruning eliminates unnecessary synapses and ensures optimal brain function. The latter activity is likely mediated *via* regulated C1q-directed complement activation on synapses that are then phagocytosed by complement receptor-expressing microglia (107). Thus, mice deficient in either *C1qa* or *C3* display defects in synaptic pruning and have abnormal brain development and abnormal behavior upon stress exposure (108). Although most of the work so far has, naturally, been performed in small animal models, it is becoming clear that complement is also ‘at the heart’ of a healthy human brain. The number of studies describing a strong association between complement system perturbations and diseases such as schizophrenia and epilepsy, disorders that are linked to altered brain development in humans, are constantly increasing. These associations are often linked to single nucleotide polymorphisms (SNPs) in the *C3*, *C4* or complement Factor H encoding *CFH* genes (109). In fact, SNPs in the *C4A* gene are currently the strongest polygenic associations with schizophrenia in humans. Similarly, patients suffering from severe depression display augmented *C3* gene expression in the prefrontal cortex (PFC). Selective C3 overexpression in the PFC of mice in a chronic stress-induced model also triggers depressive-like behavior (108). Furthermore, aberrant complement activation is connected with the induction and/or pathogenesis of a broad range of acute and chronic neurological disorders, including traumatic brain and spinal cord injuries, amyotrophic lateral sclerosis (ALS), MS, neuromyelitis optica (NMO), stroke, Alzheimer disease (AD), Parkinson disease (PD), and Huntington disease (HD) (110–117).

While the regulation of CNS development and, in consequence, cognitive function by local extracellular complement is by now well appreciated, a potential role for the complosome in the brain has not been experimentally addressed. However, as in all other tissues, metabolic regulation of the brain is critically important for its physiological function – both on the molecular and cellular level. While these processes are not yet fully understood, it is evident that neural activity and thus cognitive function depend on high energy consumption. Dynamic neural processes necessitate continuous metabolic adaptation to sustain a range of vital activities. These include providing energy for neural functions, such as plasticity, neurotransmitter synthesis, processing and recycling and also for the removal or buffering of toxic metabolic byproducts, including ROS generated during these processes (118). The complosome directs these important metabolic processes in a broad range of myeloid and lymphoid immune cells in the periphery and it is hence entirely feasible that it also partakes in the control of specialized (immune) cells in the brain. Indeed, extracellular C1q changes cholesterol metabolism in neurons and thereby protect neurons against β-amyloid-induced neuronal cell death (106). Importantly, C1q is also active intracellularly and regulates mitochondrial activity and cell death in T cells and epithelial cells (119–122). It would thus not be at all surprising if C1q serves an additional metabolic role in the brain, as part of the complosome. Overall, we hypothesize that the complosome plays a role in normal development and physiology of the CNS and/or brain – a proposal that we are actively probing in the lab.

### Non-Canonical Activities of IFN-γ in Normal Brain Function and Behavior

Interestingly, and similar to complement, cytokines that have traditionally been connected with the diseased or inflamed CNS, are now being identified as critical mediators of physiological brain function. The roles of cytokines associated with the innate immune response, such as IL-1β, IL-6 and Tumor necrosis factor (TNF)-α, in synaptic plasticity, neurogenesis and neuromodulation have been elaborately studied [for a detailed description see (123)]. However, current research established IFN-γ and IL-10, cytokines typically produced by T cells as equally important contributors to the maintenance of physiological brain function (30, 124–129). Because of the tight connection between the complosome, IFN-γ and IL-10 (see above), we will focus exclusively on the novel non-canonical roles of these two cytokines in the brain.

Due to their ubiquitous presence IFNs have been proposed to be the key messengers mediating communication between the CNS, the immune- and the endocrine systems (130). IFNs are an endogenous pleiotropic family of cytokines, which consist of the three subtypes: type I (several homolog IFN-α types and a single IFN-β in mice and men), type II (IFN-γ), and type III (IFN-λ1 to -λ4). Of these, IFN-γ is the most evolutionarily conserved between species and almost exclusively produced by T and natural killer (NK) cells (131).

**Figure 3:**
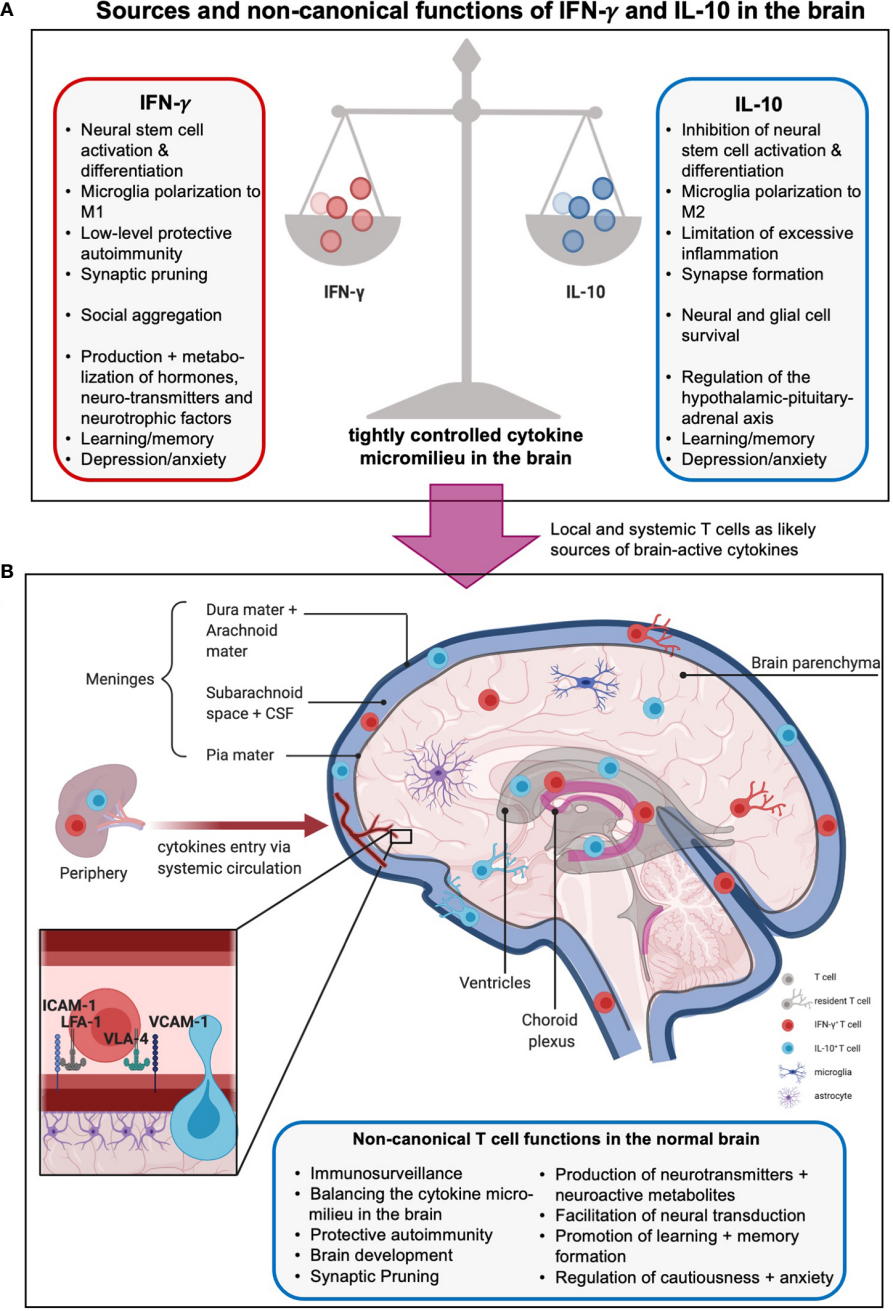
Sources and non-canonical functions of IFN-γ and IL-10 in the brain. **(A)** The cytokine milieu in and around the brain is tightly controlled to allow beneficial, hemostatic low-level autoimmunity but prevent excessive inflammation. Thus, pro-inflammatory local IFN-γ which promotes neural differentiation and synaptic pruning is restrained by local presence of anti-inflammatory, stemness preserving, and synapses formation supporting IL-10. The boxes summarize further unique and shared functions of these cytokines in metabolism, hormone regulation, and behavioral control (see text for more details). **(B)** The origins of IFN-γ- and IL-10 in the central nervous system (CNS) are not fully delineated. Sources include production by local tissue-resident cells such as microglia and astrocytes, influx from the periphery *via* a compromised blood-brain barrier (BBB), and/or, production by T cells residing in or are adjacent to the brain parenchyma, meninges, choroid plexus, circumventricular organs and ventricles. Brain-associated T cells enter these regions mainly *via* the recently discovered meningeal lymphatics, and/or extravasation through endothelial cells lining blood vessels within the CNS, and/or crossing the BBB involving an ICAM-1- or VCAM-1-mediated mechanisms (insert). Brain T cells can be resident or patrolling and mediate immunosurveillance and protection against infection but also the normal development and physiological activities of the brain, including host behavior and emotions *via* production of effector molecules (cytokines, neuro-active metabolites, neurotransmitters, etc., see text for more details). Moreover, CD4^+^ T cells have been shown to produce neurotransmitters as well as further neuromodulating agents and metabolites such as glutamate and lactate, regulate neural synapse formation and mediate neural transduction. Of note, the effects of T cells on neural functions and emotions are complex and strongly influenced by spatial and temporal context and micromilieu in the brain.

Because of its deleterious effect on tissue integrity, IFN-γ was thought to be excluded from the brain and only present after compromise of the blood-brain barrier (BBB) and/or secreted by brain-infiltrating pro-inflammatory T and NK cells during neuro-inflammation. IFN‐γ clearly contributes to demyelinating disorders, such as MS in humans and experimental autoimmune encephalomyelitis (EAE) in mice. However, mouse strains normally resistant to EAE are perplexingly susceptible to disease when either the *Ifng* or *Ifngr1* genes are mutated (132). This suggests that the biology of IFN-γ in the brain is complex and that its effects are dictated by dose and context. Indeed, physiological low level IFN‐γ in the brain protects against several mouse models, such as chemically-induced CNS demyelination (132, 133) and stab wound brain injury (134). Moreover, IFN‐γ enhances neuronal differentiation *in vitro* when directly administered to human adult neural primary progenitor cell (NPC) (124) and cell lines (125, 126) or to NPCs derived from rat and mouse embryos (127). Similarly, transgenic mice constitutively expressing low levels of IFN‐γ in the periphery display increased NPC proliferation and differentiation, improved spatial learning and increased memory performance (135) **(** **Figure 3A** **)**. Intriguingly, (T-cell-derived) IFN‐γ also modulates synaptic pruning in the context of viral infections (136), a process that might also, at least in part, be mediated by complement since IFN‐γ induces elevated expression of C3, a factor that mediates synaptic pruning, in human astrocytes (137, 138).

The exact mechanisms underlying these activities of IFN-γ are a current research focus, but one potential route may be *via* exertion of control over hormones and/or neurotransmitters. IFN‐γ in the brain regulates expression and/or metabolism of several hormones, such as adrenocorticotropic hormone (ACTH), growth hormone (HGH), and prolactin (PRL, which is also a central regulator of lipid metabolism) (130, 139), brain-derived neurotrophic factor (BDNF) (134), and neurotransmitters such as serotonin (140, 141) and noradrenaline (142). Consistent with these findings, IFN-γ-deficient mice mount attenuated hormonal and dopaminergic responses when exposed to chronic stress (143). IFN-γ may also control local metabolic pathways as the indolamine 2,3-dioxygenase-1 (IDO1) metabolism pathway in mice overexpressing IFN-γ is elevated (144, 145). Overactive IDO1 dysregulates L-tryptophan metabolism by favoring production of kynurenine and its neurotoxic metabolites 3-hydroxykynurenine (3-HK) and quinolinic acid (QUIN), thereby decreasing the bioavailability of L-tryptophan for serotonin production. Overall, IFN-γ-mediated neuroprotective and/or neuromodulating effects deeply impact behavior: mice overexpressing IFN-γ display anhedonic and depression-like behavior (145) and animals lacking IFN-γ exhibit heightened anxiety (143, 146). The centrality of this cytokine in brain biology across evolution and species is further underpinned by that fact that rodents, fish, and flies all elevate IFN-γ-driven gene signatures in specific social contexts and that mice lacking IFN-γ production exhibit social deficits in groups (30). In the latter, direct cerebrospinal IFN-γ injection or repopulation of the meninges with IFN-γ-producing T cells reverted the animals’ social behavior to normal by increasing inhibitory γ-aminobutyric acid (GABA)-ergic currents and preventing hyperexcitability in the prefrontal cortex (30).The murine connection between IFN-γ and behavior also extends to humans and seems to be particularly important in the context of depression. Thirty to 70% of patients treated with IFNs (to, for example, treat immunodeficiency) develop depression as a major side effect (147). Furthermore, a single nucleotide variant in the *IFNG* gene was recently reported to elevate the risk of depression in the context of clinical IFN-α treatment (148). Conversely, several studies have reported pathologically elevated levels of circulating IFN-γ among depressed patients (149–151). One of the fundamental questions regarding the role of IFN-γ in the CNS centers on the origin of this cytokine. The three most likely sources, which are not mutually exclusive, include, 1) resident CNS cells, such as microglia and astrocytes, directly producing IFN-γ in response to stimulation (152, 153); 2) peripheral IFN-γ infiltrating the brain through a compromised BBB (154, 155); and/or, 3) resident or parenchyma-adjacent T cells producing IFN-γ in response to stimulation (31, 156) **(** **Figure 3B** **)**. Since the complosome, and particularly C3, is intimately connected with IFN-γ production, it is likely that intracellular C3 could play a role in all three of these possible scenarios.

### Non-Canonical Activities of IL-10 in Normal Brain Function and Behavior

Whereas IFN-γ in the brain is somewhat of a double-edged sword, IL-10 has a more dependable, beneficial, impact (157–161), consistent with the classic immunosuppressive and housekeeping role of IL-10 in immunity (162, 163). *Il10^–/–^* mice have more depression and helplessness-like behavior in forced-swim tests; this can be normalized by injection of IL-10. Conversely, mice overexpressing IL-10 have reduced depression (164). The underlying mechanism is not clear but existing data indicate that IL-10 negatively regulates the hypothalamic-pituitary-adrenal (HPA) axis in both mice and humans. The HPA axis regulates glucocorticoid feedback sensitivity and, as such, is the body’s central stress response system (165). The HPA is consistently overactive in depressed patients (166). IL-10 also maintains neural progenitors in an undifferentiated state, impairs neuronal differentiation and, collectively, controls neurogenesis (128). It also promotes synaptic formation (129) and, consequently, learning and memory formation, at least in mice (129, 167). The sources of IL-10 in the brain have not yet been delineated. However, the range of cells capable of producing IL-10 is quite broad and includes B cells, macrophages, dendritic cells, neutrophils and eosinophils (168), as well as astrocytes and microglia (169).

### Central Nervous System-Adjacent and -Resident T Cells in the Healthy Brain

The prevailing opinion has traditionally been that the healthy CNS and especially the brain are immune privileged spaces, sealed by a tight BBB (170). Thus, any immune cell found within the CNS or brain was thought to indicate neuro-inflammation. Recent discoveries dramatically challenged this understanding, and it is now accepted that sites adjacent to the healthy brain, in particular the meninges, the choroid plexus, the circumventricular organs and ventricles, the perivascular areas, and the brain parenchyma itself, are populated with immune cells (31, 170, 171). Moreover, T cells are the predominant cell type observed and it is, thus, likely that they are one of the major sources of IFN-γ and/or IL-10 in the brain (31). Here, we will focus on the non-canonical roles of T cells in support of normal brain physiology (**Figure 3B**). Such ‘brain-associated’ T cells enter these regions mainly *via* the recently discovered meningeal lymphatics and/or *via* extravasation through endothelial cells lining blood vessels within the CNS. Importantly, transmigration of T cells *via* the endothelium into key brain adjacent tissues and spaces involves T cell expression of LFA-1 or very late antigen (VLA)-4 and their engagement with their cognate receptors on endothelial cells, ICAM-1 or VCAM-1, respectively (172). As discussed above, the LFA-1 – ICAM-1 axis is central to C3 loading of T cells. It is thus plausible that similar events enable complosome-mediated T cell functions in brain adjacent areas – as previously shown for the lung (27).

We are only beginning to unravel the functions of brain-associated T cells. Indication is strong though that T cells in these regions can be both sessile (and thus truely resident) or patrolling; further, they seem involved in both provision of immunity as well as physiological regulation of brain function (171). While much effort has been put into defining the nature of T cells in the brain parenchyma (see below), a recent study elegantly showed that the temporal presence of CD4^+^ T cells in the mouse and human brain is critically required for normal organ development (31, 171). Failure of T cell (conventional and regulatory T cells) migration from the blood to the brain around birth leads to the arrest of transcriptional maturation of microglia and defective neural pruning. In consequence, mice in which T cell migration to the brain has been ablated or that lack expression of major histocompatibility complex (MHC) II (which normally fosters interactions between T cells and microglia) are anxious and depressed (31). Interestingly, while both naïve cells and activated T cells cross the CNS in a transient manner, only activated, antigen-experienced T cells are retained within the brain parenchyma for prolonged periods of time (31, 171, 173). Since complosome induction is a hallmark of activated (Th1) T cells and generally of T cells in tissues, this may again indicate that the complosome could be at play here (27). The requirement for T cell activation or exposure to antigen to establish brain residency raises several questions. For example, are only specific T cell subsets enriched and, if so, what is the cognate antigen of such T cells? Finding answers to these questions is a principal focus in the field.

Both CD8^+^ and CD4^+^ T cells have been described in the brain, expressing markers commonly associated with tissue-residency (31, 174–177). CD8^+^ T cells are mostly represented by slowly-dividing, self-replicating and neuroprotective tissue-resident memory (T_RM_) CD103^+^ cells (174, 175, 177). They are dependent on antigen presentation *in situ* and thus were initially believed to arise locally upon brain insult and/or infection (175). Accumulating data indicates, however, that they can migrate into the brain from the periphery even in the absence of infection. The neurotropic cues of peripherally activated CD8^+^ T cells are currently under examination (177–179). A recent in-depth study performed using mice and human brain tissues found high numbers of effector-like CD4^+^ T cells at this location, with about 8% of T cells having a Treg phenotype (31). Moreover, the conversion rate from a transient to a resident phenotype was much faster in the effector-like T cells when compared to Tregs at this location. This observation is in stark contrast to the prevalent and intuitive assumption that only regulatory and anti-inflammatory T cells are allowed in the brain, while conventional, pro-inflammatory subsets should be excluded to prevent toxic neuroinflammation. However, as detailed above, pro-inflammatory effector cytokines, such as IFN-γ, can be beneficial to the CNS. Indeed, it has been widely reported that conventional, IFN-γ-producing and autoreactive T cells may actually protect against detrimental neuroinflammation, a concept coined as ‘protective autoimmunity’ (180, 181). Such protective low-level autoimmunity may mechanistically involve control of CNS self-antigens by local T cells (182, 183). Although IL-17–producing Th17 cells are critical modulators of neuroinflammation and data about a potential homeostatic role for Th17 cells in the brain are sparse, there are few pointers indicating that they are also not all bad. For example, γδ T cells in the meninges of mice constitutively produce IL-17, which acts on glia to produce the nerve growth factor BDNF and thus supports synaptic plasticity and spatial memory acquisition (184). Similarly, sociability in mice seems to depend on, or is increased by, IL-17A in the CNS (185).

Overall, these data indicate that presence of T cells producing low IFN-γ (and possibly IL-17) in the CNS/brain is physiological and neuroprotective. Of course, the degree of inflammation needs to be tightly controlled to prevent excessive, tissue-damaging and deleterious effects (181) – a concept which coincidently also holds true for the complement system. Control over micro-inflammation in the brain is in large parts achieved by engaging several mechanisms that ensure an overall anti-inflammatory environment. These involve the presence of anti-inflammatory and IL-4 producing Th2 cells (186) and astrocytes actively restraining T cell proliferation and IFN-γ production (187, 188). Given that the complosome is a central controller of the magnitude and duration of Th1 responses, by mediating switch of Th1 cells from production of pro-inflammatory IFN-γ into anti-inflammatory IL-10 (26, 72, 73), we posit that such complosome-instructed human Th1 cells are uniquely equipped to promote low-levels of autoinflammation with an integrated secure lid provided by an IL-10-driven control program. Indeed, Tr-1 like Foxp3^+^ (Forkhead Box P3) cells, which are phenotypically similar to complosome-controlled contracting Th1 cells, play a key role in the restriction of neuroinflammation (189). Additionally, immunization of mice with the neural self-antigen myelin basic protein (MBP) induces conversion of conventional CD4^+^ T cells into IL-10 secreting, neuroprotective CD4^+^ T cells *via* a mechanism involving mTOR inhibition (190), a machinery that is also engaged by the complosome to induce Th1 to IL-10 switching (26).

In summary, both CD4^+^ and CD8^+^ T cells can have neuroprotective properties, surprisingly mediated by their pro-inflammatory effector cytokines. Their beneficial homeostatic activities in the CNS are sustained *via* a network of immune and structural cells that cooperate to curb detrimental local increases in T cell-derived inflammatory factors (183, 191, 192). We suggest that a second important layer of such control is exerted by the T cell intrinsic complosome.

## Postnatal Behavioral Control by T Cells

As discussed above, perturbations in T cell activity during CNS and/or brain development lead to deviations from normal cognitive function and induce changes in behavior. However, there is now also accumulating evidence indicating a significant role for T cells in regulation of behavior postnatally (30, 32, 193–196) (**Figure 3** **)**. This is a relatively nascent research area and the exact nature of the T cell subsets and the effector mechanisms mediating observed behavioral changes have not been fully elucidated. However, one emerging model is that of a metabolic communication between T cells and the brain.

### Learning and Memory

The ability to learn and to memorize information is central to life and the ability of the individual to actively integrate into society. These critical cognitive functions are based on remodeling of neural circuits, promotion of memory consolidation, hippocampal long-term potentiation (LTP) and neurogenesis. Hippocampal neurogenesis is required for spatial and non-spatial learning and memory (197) and is abnormal in mice without lymphocytes (severe combined immune deficiency (SCID), nude, and *Rag1^–/–^* mice). Accordingly, these animals all have impaired spatial learning and memory capabilities which can be restored in all cases upon transfer of CD4^+^ T cells, demonstrating an active and continuous role for T cells in these processes (193, 196). Similarly, age-related memory impairment in old mice can be restored by homeostatic T cell proliferation (195). Likewise, transgenic mice with T cells specific for a brain-confined antigen exhibit generally improved learning and memory (196). The T cell-derived factors supporting memory and learning are not fully defined but include IL-4 (198, 199), lactate and glutamate. These metabolites are both central to normal hippocampus activity and spatial learning and memory retention and are thought to be controlled by the metabolic coupling of astrocytes and neurons (200). Incidentally, Th1 cells also generate and specifically secrete high levels of both lactate and glutamate, driven by complosome-supported glycolysis and glutaminolysis (72).

### Emotions and Social Behavior

Social behavior within given norms allows group living which provides vital benefits to individuals, including shared (food) resources and companionship. As discussed above, meningeal T cells can control social aggregation and IFN-γ emerges as a specific molecular link between T cells and the neural circuits underlying social behavior (30). Group living, however, also comes with the likelihood for conflict and anxiety. The latter is one of the best investigated emotions in animal models and the amygdala plays a critical role in anxiety development and control (201). T cell deficient mice not only have learning defects but also display attenuated anxiety behavior (202, 203), suggesting a physiological role for T cells in the mediation of cautiousness and, in consequence, possible pathological anxiety. However, *Rag1* ^–/–^ mice (which have no T cells) have also shown to exhibit augmented instead of reduced anxiety-like behavior, which is rescuable by T cell transfer in distinct anxiety models (191). This indicates that specific anxiety triggers may evoke distinct responses in different mouse strains. A further drawback of several of these studies is that it is not always possible to conclusively determine whether observed effects are due to prenatal or postnatal absence of T cells. Nonetheless, in most settings, a clear positioning of T cells within the brain is required for an impact on mood and behavior. This has elegantly been demonstrated in a model of lymphocyte transfer from chronically social-defeated mice into healthy recipient mice, who then exhibited suppressed anxiety, contingent on T cells entering the choroid plexus in an ICAM-1–dependent fashion (204, 205).

As for learning and memory, the T-cell derived modulators of mood and behavior are mostly still under investigation, but also have a clear metabolic component: physical stress-induced leukotriene (LT) B4 triggers mitochondrial fission in mouse CD4^+^ T cells and interferon regulatory factor (IRF)-1 activation culminating in increased purine synthesis. Consequently, glucose is redirected toward the pentose phosphate pathway (PPP) and the produced metabolite and oligodendrocyte growth factor xanthine then increases anxiety-like behavior (32). Also, normal glycolysis-derived lactate (of which Th1 cells are a major source) has long been known to have anti-depressant activities (206). Similarly, T cell-derived IL-10 appears to impact behavior, because protective immunization with MBP in mice involves a T cell-mediated reduction in nerve cell activity *via* IL-10, permitting injured nerve cells to preserve energy for faster recovery and stress-coping (181). In all, currently available data firmly place T cells and their cytokines and metabolites as contributors to mood and behavior modulation. One of the major activities of the complosome in T cells centers around the induction of a series of transporters that ensure nutrient influx needed for T cell (effector) function as well as control of metabolic enzymes (18). Intriguingly, the complosome also regulates the secretory machinery in T cells which controls the export of growth factors and metabolites (unpublished data). It is hence ideally placed to mediate the metabolic cross-communication between T cells and the brain parenchyma.

## A Non-Canonical Evolutionary View on the Complosome-Metabolism-IFN-γ Axis

**Figure 4:**
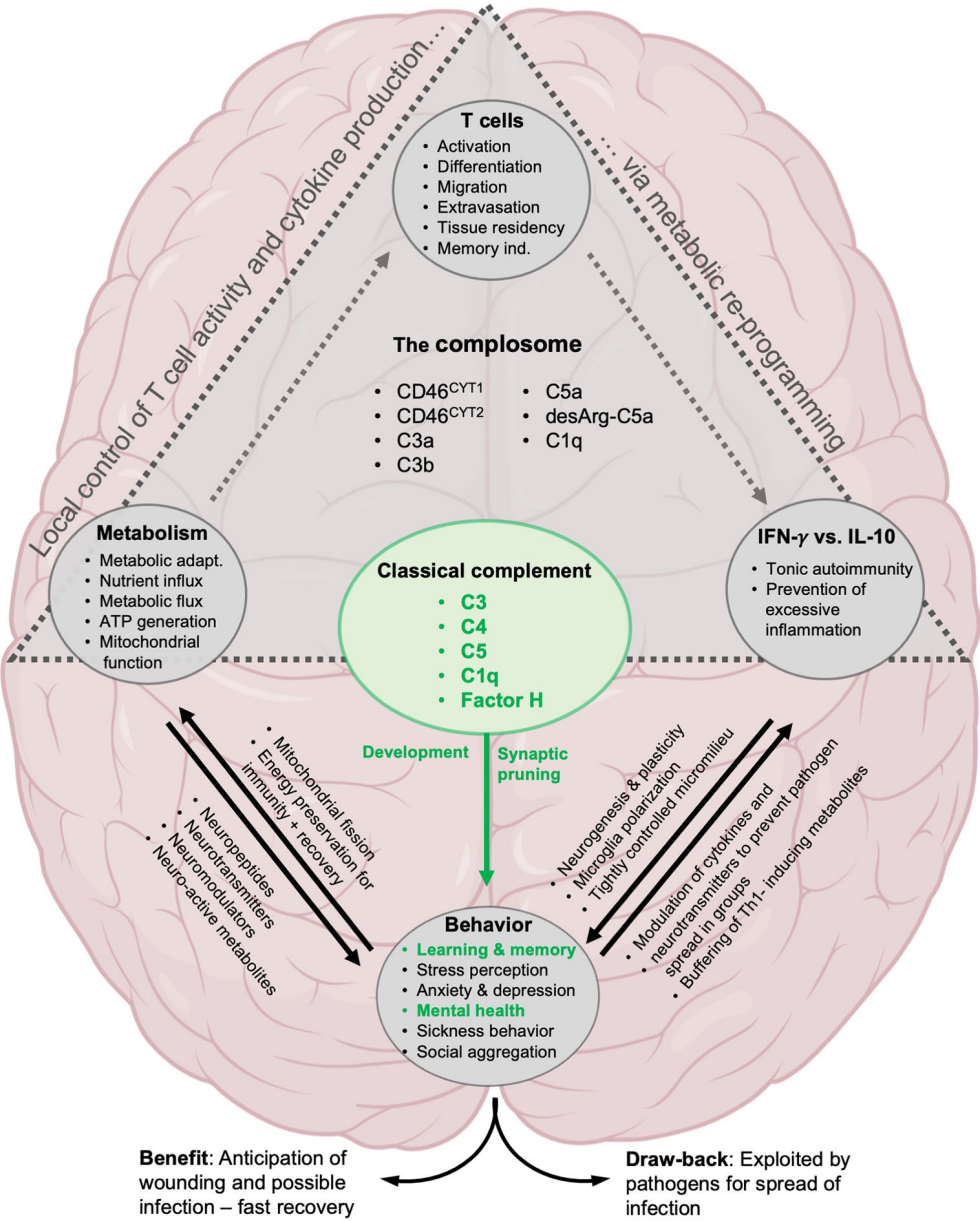
Proposed involvement of the complosome in behavioral control. The direct effect on brain development by classical complement system is well established: C1q and C3 mediate synaptic pruning, neurogenesis, cognitive functions and are important for mental health (green text). Likewise, the role of complement and the complosome in particular in the control of local T cell responses *via* metabolic reprogramming has been well described (gray triangle, see text for details). We propose that the complosome is heavily involved in most of the known effects of T cells, metabolism and cytokines (IFN-γ and IL-10) on neural functions underlying cognition and behavior.

The close connection between complement and IFN-γ has always been viewed as a logical partnership between the most potent innate arm of immunity and one of the most effective cytokines in the fight against infection. However, in light of the evolutionary age and emerging non-canonical functions of both partners, our laboratory now considers another, or additional, reason for the early emergence and successful survival of the complosome/metabolism/IFN-γ axis (**Figure 4**). This idea is based on the view that the CNS and our immune system are not separate entities engaging in crosstalk during host development and/or infection but that they are rather inter-related systems required to drive social capability and behavior needed for higher order organisms to survive and thrive. In the following section, we will introduce the two concepts underlying our proposition, the concept of anticipatory neuro-immune reactivity to environmental threat, and the concept of evolutionary co-dependence of behavioral and immunological activities. Both propositions rely on the assumption that immune cells, and by extension their cytokines, are fundamental information mediators between the CNS and other specialized tissues.

Metazoan evolution is the progressive specialization of single cell and tissue function from a shared and pluripotent precursor. In the course of this process precursor cells split eventually into two branches: parenchymal cells (cells constituting the tissues itself) and accessory cells (mainly stromal, endothelial, and immune cells) to ensure optimal division of labor in the host body. Parenchymal cells constitute the framework for each tissue, are immobile, and specialized in their characteristic tissue function. Accessory cells, on the other hand, support homeostasis across tissues and mediate the response to incoming environmental signals (207). Thus, accessory cells are dynamic, more highly plastic, and responsive to environmental signals (207). Among accessory cells, immune cells are the highly mobile fraction and can, upon cue, reach most tissues rapidly *via* the circulatory and lymphoreticular systems. This makes them the ideal mediators and synchronizers between individual cells, tissues, organs and the CNS, a communication needed to initiate and execute whole-organism responses. In the context of infection this entails coordinating a direct anti-microbial response in the affected tissue, directing the metabolic adaptation which balances cell/tissue energy consumption toward infection control, and inducing host behavioral adaptations, such as avoidance of the infectious source and social withdrawal to prevent spread of infection. Upon pathogen clearance, the very same immune cells – e.g. contracting Th1 cells (26) – are equally involved in confining inflammation, orchestrating tissue-regeneration and promoting behavioral normalization. This holistic view of T cell activity aligns with their capability to generate a large range of non-canonical, non-immunological signaling molecules, including growth factors, hormones, neurotransmitters, neuromodulators and neurotrophic factors involved in CNS regulation (208–210). Moreover, T cells express receptor for all of these mediators themselves (211, 212), indicating their nodal role in the shared and multi-directional communication between the immune, hormonal and neural systems. The bidirectionality of this cooperation suggests that the neural system, which constantly monitors the environment for potential threats, can also signal danger to the immune system to (pre-emptively) allow it to engage in an alerted and poised state. Indeed, such an anticipatory combined neuro–immune response likely provided a strong evolutionary advantage during interactions with peers, predators, and pathogens among early humans (213, 214). For example, social conflict, aggression and social isolation rendered our ancestors vulnerable to attacks from predators or rivals, which in turn increased the probability of being physically wounded and succumbing to infection (213, 214). Thus, a danger perception-mediated heightened readiness state of the immune system would be accompanied during true pathogen encounter by faster induction of immunity and sickness behavior (anhedonia), which conserves energy for pathogen removal and wound healing. Intriguingly, this beneficial interconnection of neural and immune responses has been established early on in evolution as it can already be observed in the nematode *Caenorhabditis elegans*. In *C. elegans*, the neurotransmitter serotonin directly regulates protective immune responses at epithelial barriers (215, 216) and also mediates neuron-induced avoidance behavior and olfactory learning circuits that effectively translate into decreased infection susceptibility (217, 218). Importantly, the sensory mechanisms mediating avoidance behavior and olfactory learning also induce expression of immune-protective genes, some of which are not only involved in direct immune defense but also contribute to subsequent behavioral conditioning (219). While the role of IFN‐γ has not yet been explored, IL-17 is a key regulator of *C. elegans* neuro-modulatory sensing and behavior (220).

Another evolutionary perspective was suggested recently by Kipnis *et al*. Based on the evolutionarily conserved role of IFN‐γ in directly driving social behavior and group connectivity ( (30, 208), and above), this group speculates that IFN‐γ initially developed as a behavioral mediator supporting individual sociability – and was then hijacked by pathogens to promote inter-individual spreading (30). IFN-γ subsequently gained additional antimicrobial functions to counteract this very same microbial abuse in a co-evolutionary “arms-race” between pathogens and host defense. In fact, recycling or expansion of molecular functions for additional purposes is an age-old process as evolutionary pressures drive divergence of shared precursor gene and proteins toward specialized functions to accommodate increasing complexity of diversification in evolving species. The hypothesized hijacking of the beneficial effects of IFN-γ on social behavior by pathogens is supported by the fact that up to 10% of the human genome consists of endogenous retrovirus (ERV) DNA. These are believed to originate from ancient viral infections that accumulated in the host over time (221). Although ERVs cannot replicate, they do impact host behavior. For example, aberrant expression of the murine ERVs *MMERVK10C* and *IAP1* alters expression of nearby genes (and non-coding RNAs) and result in behavioral alterations (222). In humans, long terminal repeats (LTRs) of the MER41 family of ERVs are enriched in consensus binding sites for IFN‐γ-driven TFs, such as STAT1 and IRF1, and can further amplify expression of IFN-γ-inducible genes (223). Intriguingly, promoter regions of intellectual disability-associated genes contain a high abundance of MER41 sequences (224) and increased levels of ERV mRNA have been associated with psychiatric disorders such as schizophrenia (223). Moreover, the MER41-cognition pathway has also recently been linked to induction of Forkhead Box P2 (FOXP2), a central TF involved in human speech evolution. Pathogens are the main selective pressure through human evolution (225) and past interactions, particularly with IFN-γ response-evoking pathogens, have elicited widespread and coordinated genomic responses that often underly polygenic selection (226). Thus, viruses may not only have introduced a pathogen-fighting facet to the original developmental/behavioral mission of IFN-γ but may also have promoted changes in the DNA landscape that ultimately helped us to evolve unique features, such as speech and self-consciousness (224).

Interestingly, a few years ago, we put forth a similar non-classical concept for the evolution of the complosome. While most view the complement system expressed by the liver and operating in circulation to combat pathogens as evolutionarily first, we suggest that complement evolved initially as an intracellular, homeostatic system, the complosome. The main function of the complosome was, and remains, regulation of nutrient availability (influx/efflux) and basic cellular metabolism in an mTOR-dependent manner. This close connection to the mTOR machinery established the conditions that enabled the special relationship between the complosome and the energy-intense production of IFN-γ. In theory, this connection could thus place the complosome-metabolism-IFN-γ axis as an early evolutionary driver of CNS/brain development and behavior (**Figure 4**). During evolution from single to multicellular organisms, and under pressure from infectious agents, complement acquired additional extracellular functions and transformed in parts into the fluid-phase, anti-pathogenic, system that it is mostly known for today (227).

Of course, our proposed novel role for the complosome in general tissue biology, and the CNS in particular, is currently purely speculative and awaits experimental validation. This will take time because studying the complosome requires the generation of new reagents and models. For example, T cell-intrinsic C3 exists in different structures with distinct post-translational modifications when compared to liver-derived C3 and, thus, does not often cross-react with commonly used anti-C3 antibodies (2, 122). Furthermore, there are substantial differences between the exact composition of the complosome [for example, mice lack CD46 (60)] and its functional engagement with other intracellular PRRs (for example, NLRP3 in mouse T cells is not required for Th1 induction) between species. These hurdles make studying the complosome *in vitro* but specifically probing it using *in vivo* studies with human correlates currently complex. Nonetheless, we suggest that understanding the potential role of the complosome at the neuro-immune interface could provide new avenues to first understand and then possibly treat mental disorders.

‘The true delight is in the finding out rather than in the knowing’ (Issac Asimov, 1920 – 1992)

## Author Contributions

Conceptualization, writing—original draft, and revision: NK and CK. All authors contributed to the article and approved the submitted version.

## Funding

Work in the Complement and Inflammation Research Section (CIRS) is financed by the Division of Intramural Research, National Heart, Lung, and Blood Institute (NHLBI), NIH Clinical Center Work at CIRS is supported by the NIH Intramural Research Program.

## Conflict of Interest

The authors declare that the research was conducted in the absence of any commercial or financial relationships that could be construed as a potential conflict of interest.

## Abbreviations

3-HK, 3-hydroxykynurenine; AAs, amino acids; ACTH, adrenocorticotropic hormone; AD, Alzheimer’s disease; AP-1, activation protein 1; APCs, antigen-presenting cells; ALS, amyotrophic lateral sclerosis; ATP, adenosine triphosphate; BBB, blood–brain barrier; BC, BDNF, brain-derived neurotrophic factor; before Christ; C3aR, C3a receptor; *C. elegans, Caenorhabditis elegans;* CFH, complement factor H; CSF, cerebrospinal fluid; CNS, central nervous system; CTL, cytotoxic T cell; CTSL, cathepsin L; DAMPs, danger-associated patterns; EAE, experimental autoimmune encephalomyelitis; ER, endoplasmatic reticulum; ERV, endogenous retrovirus; FOXP, Forkhead Box P; GABA, gamma-aminobutyric acid; GH, growth hormone; GLUT-1, glucose transporter 1; GPCRs, G protein-coupled signaling receptors; HD, Huntington’s disease; HPA, hypothalamic-pituitary-adrenal; ICAM-1, intercellular adhesion molecule 1; IDO1, indolamine 2,3-dioxygenase-1; IFN, interferon; IL, interleukin; JAK, janus kinase; LAD-1, leukocyte adhesion deficiency type 1; LAT-1, large neutral amino acid transporter; LFA-1, lymphocyte function-associated antigen 1; MAC, membrane attack complex; MBP, myelin basic protein; MCP, membrane cofactor protein; MHC, major histocompatibility complex; MMP, metalloproteinase; MS, multiple sclerosis; mTOR, mammalian target of rapamycin; mTORC1, mTOR complex 1; NAD^+^, nicotinamide adenine dinucleotide; NK cell, natural killer cell; NLRP3, NLR family pyrin domain containing 3 protein; NMO, neuromyelitis optica; NPC, neural primary progenitor cell; OXPHOS, oxidative phosphorylation; PAMPs, pathogen-associated patterns; PFC, prefrontal cortex; PD, Parkinson’s disease; PKM2, pyruvate kinase M2; PPARs, peroxisome proliferator-activated receptors; PRRs, pattern-recognition receptors; pSMAC, peripheral supramolecular activation cluster; QUIN, quinolinic acid; RA, rheumatoid arthritis; RLRs, RIG-I like receptors; ROS, reactive oxygen species; SARS-CoV2, severe acute respiratory syndrome coronavirus 2; SLE, systemic lupus erythematosus; SNPs, single nucleotide polymorphisms; STAT, signal transducer and activator of transcription; TGF, transforming growth factor; Th, T helper subset; TLRs, toll-like receptors; TNF, Tumor necrosis factor; Tregs, regulatory T cells; Tr1 cells, T regulatory 1 cells; T_RM_ cells, Tissue-resident memory cells; VCAM-1, vascular cell adhesion molecule 1; VLA-4, very late antigen 4.
